# Confronting emerging infections: lessons from the smallpox eradication campaign.

**DOI:** 10.3201/eid0403.980318

**Published:** 1998

**Authors:** W H Foege

**Affiliations:** Emory University, Atlanta, Georgia, USA.


### Confronting Emerging Infections: Lessons from the Smallpox Eradication Campaign

**William H. Foege**
Emory University, Atlanta, Georgia, USA

Ralph Waldo Emerson in 1860 said, “We learn geology the day after the earthquake.” Traditionally, the world learns prevention the day after the epidemic. Today, we have the responsibility of preparing for the prevention and control not only of known but also unknown conditions. Eradication is a focused field exercise in which approaches have been tested and from which public health lessons can be learned.

### Lessons from Eradication

### Calculated Risks

It is clear, in retrospect, that we didn’t know how to eradicate smallpox when the eradication effort began. Thirty years ago, in the middle of the smallpox campaign in West and Central Africa (charged with ending transmission in 20 countries in 5 years), we tried a new strategy, converting from mass vaccination to surveillance and containment. Although we were 1 1/2 years into the campaign when the strategy shift occurred, we still reached the goal of zero cases on time and under budget. The lesson is that we do not have the luxury of waiting until we know everything before doing something. We are always called upon to make decisions with insufficient information and make corrections midcourse.

### Interdependence

Disease eradication campaigns illustrate the value of working as global citizens rather than as a collection of national programs. First promoted by the Soviet Union in 1958, smallpox eradication did not get the approval of the World Health Assembly until 8 years later in 1966, when it became a joint proposal of the Soviet Union and the United States. If we could form this alliance during the cold war, how many alliances can we form now? No country alone can prevent or control emerging infections.

### Knowledge

We did not understand the limitations of smallpox transmission; we knew nothing about fetishes or the role of nomads. As organisms, the environment, people, and tools change, programs must change. Appropriate response requires good epidemiologic analysis. The epidemiology, in turn, can be no better than the facts assembled. Knowledge is dependent on the information system; in public health, the surveillance system forms the foundation of knowledge.

### Vision

With eradication, the vision is no more cases. With emerging infections, the vision is rapid, appropriate, effective response, being prepared to protect the world because you are ready to act.

### Performance

With eradication, to get global support, we must demonstrate that a disease can be eliminated from a geographic area. With emerging infections, the value of surveillance (for making decisions, for deciding on interventions) must be demonstrated.

### Humility

With all our experience, we have not gone far on the road to eradicating disease. This knowledge keeps us humble. We have trouble outthinking a virus. Even smallpox humbled us until the very end. That virus seemed to have a better understanding of nature, human behavior, and ways to achieve immortality than the entire smallpox eradication team. The emergence and reemergence of infections must be approached with humility.

### Enemies

Some anthropologists think conflict is not only inevitable but needed. Will Durant once doubted the world could ever combine forces without fear of an alien invasion. Perhaps disease could be used as a surrogate enemy? Emerging infections are a powerful common enemy well suited as a global challenge.

### Focused Energy

Energy focused on a specific end can also build infrastructure. Energy focused on eradication improved infrastructure. Surveillance, logistic systems, evaluation, field teams, and cluster sampling are concepts used during eradication that are now part of primary health care.

### Optimism

The pessimists and cynics were not just wrong with smallpox; they were harmful. They diverted attention, generated doubts in those who could provide resources, invented problems far beyond the vast array of existing ones. Even though negative news can be of value, their usefulness is limited. Large problems should be approached with optimism.

### Conclusions

Nine hundred years ago, building inventions converged and reached a peak, leading builders and architects of the time to try ever bolder structures. Cathedrals were built that in turn led to new innovations. For several hundred years Europe was rewarded not only with cathedrals but also with better building techniques for all structures. The infrastructure changed. Historians, in a thousand years, will look on the public health cathedrals that resulted from better building materials in a period of 75 years, from the mid-20th century until the early 21st century. The control and eradication of infectious diseases that once caused great trepidation produced better diagnostic systems, treatment, and vaccines, the elements with which to strengthen and improve the public health system and confront new disease challenges.

